# Isotope Substitution Effects on the Magnetic Compass Properties of Cryptochrome-Based Radical Pairs: A Computational Study

**DOI:** 10.1021/acs.jpcb.2c05335

**Published:** 2023-01-20

**Authors:** Gediminas Jurgis Pažėra, Philip Benjamin, Henrik Mouritsen, P. J. Hore

**Affiliations:** † Department of Chemistry, University of Oxford, Oxford OX1 3QZ, U.K.; ‡ Institut für Biologie und Umweltwissenschaften, Carl-von-Ossietzky Universität Oldenburg, Oldenburg 26111, Germany; § Research Centre for Neurosensory Science, University of Oldenburg, Oldenburg 26111, Germany

## Abstract

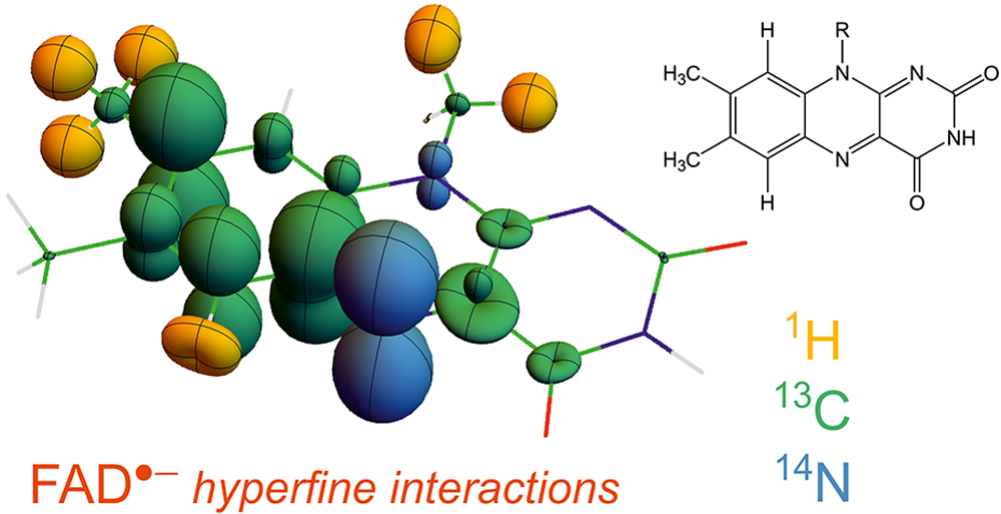

The biophysical mechanism of the magnetic compass sense of migratory songbirds is thought to rely on the photochemical reactions of flavin-containing radical pairs in cryptochrome proteins located in the birds’ eyes. A consequence of this hypothesis is that the effect of the Earth’s magnetic field on the quantum yields of reaction products should be sensitive to isotopic substitutions that modify the hyperfine interactions in the radicals. In this report, we use spin dynamics simulations to explore the effects of ^1^H → ^2^H, ^12^C → ^13^C, and ^14^N → ^15^N isotopic substitutions on the functioning of cryptochrome 4a as a magnetic direction sensor. Two main conclusions emerge. (1) Uniform deuteration of the flavin chromophore appears to be the best way to boost the anisotropy of the magnetic field effect and to change its symmetry. (2) ^13^C substitution of three of the 12 flavin carbons, in particular C4, C4a, and C8α, seems to be the best recipe for attenuating the anisotropy. These predictions should give insight into the factors that control the magnetic sensitivity once spectroscopic techniques are available for measuring magnetic field effects on oriented protein samples.

## Introduction

Migratory songbirds have a remarkable ability to use the direction of the Earth’s magnetic field to help them navigate between their breeding and wintering grounds.^1,2^ The biophysical mechanism of this light-dependent magnetic compass is uncertain but seems to involve magnetically sensitive photochemical reactions within photoreceptor cells in the retina.^3−7^ The most likely magnetoreceptor is Cry4a, one of the six known avian cryptochrome (Cry) proteins, in which short-lived radical pairs can be formed by the passage of an electron along a chain of four tryptophan (TrpH) residues to the photoexcited flavin adenine dinucleotide (FAD) chromophore in the center of the protein.^3,8−13^ In support of this proposal, flavin-tryptophan radical pairs, [FAD^•–^TrpH^•+^], in purified Cry4a from the migratory European robin (*Erithacus rubecula*, *Er*) have recently been shown to be sensitive to weak applied magnetic fields.^14,15^ However, it has yet to be demonstrated that Cry4a has the same photochemistry *in vivo* or that it satisfies other requirements for a viable magnetic direction sensor. Another possibility, for which there is currently less evidence, is that the magnetic sensitivity *in vivo* originates in a different radical pair, formed during the dark recovery of a photochemically reduced state of the protein.^16−18^ There has also been some discussion of Cry1a as an alternative to Cry4a, even though it does not bind FAD strongly *in vitro*.^19−21^

It is well established that radical pair reactions can be influenced by weak magnetic fields when certain chemical and physical conditions are satisfied.^3,22−25^ One of the most important in the context of magnetoreception is that, in at least one of the radicals, the unpaired electron must have magnetic hyperfine interactions with one or more atomic nuclei such as ^1^H and ^14^N.^3^ These interactions, which drive coherent interconversion of the singlet and triplet electronic states of the radical pair, determine to a large degree the effect of a weak applied magnetic field on the yields of the reaction products. If, as is usually the case for organic radicals, the hyperfine interactions are anisotropic, the radical pair can form the basis of a magnetic direction sensor. Both FAD^•–^ and TrpH^•+^ radicals satisfy these conditions, and indeed, the ^1^H and ^14^N hyperfine interactions in FAD^•–^ seem to be near optimum for magnetic compass sensing.^26^

A consequence of the fundamental role of hyperfine interactions in the spin dynamics of radical pairs is that isotopic substitution can in principle be used to provide insight into the factors that control the magnetic sensitivity.^27,28^ Replacing one isotope of an element by another either changes the nuclear magnetic moment and therefore the hyperfine interaction (e.g., ^1^H → ^2^H, ^14^N → ^15^N) or introduces a hyperfine interaction where none existed before (e.g., ^12^C → ^13^C). In this report, we use spin dynamics simulations to explore the effects of H, C, and N isotopic substitution on the operation of Cry4a as a compass magnetosensor. Results for both [FAD^•–^TrpH^•+^] and [FAD^•–^Z^•^] radical pairs, hereinafter abbreviated to FAD-Trp and FAD-Z, are presented (Z^•^ is a hypothetical radical with no hyperfine interactions). The aim is to determine patterns of isotopic substitution that could be interesting for future *in vitro* measurements of anisotropic magnetic field effects on oriented proteins using optical spectroscopic methods.^14,29−32^

## Methods

The spin dynamics of FAD-Trp and FAD-Z radical pairs were simulated as described in ref (33), using a density matrix master equation to account for the relevant magnetic interactions and recombination kinetics. Singlet and triplet radical pairs were assumed to react spin-selectively with equal rate constants, *k* = 10^5^ s^–1^, to give distinct products.^34^ Reaction product fields were calculated as a function of the direction of an external Earth-strength (49 μT) magnetic yield. The dipolar (*D*) and exchange (*J*) interactions in FAD-Trp were taken from a preliminary analysis of electron paramagnetic resonance data obtained from an *Er*Cry4 mutant in which the fourth (terminal) tryptophan of the Trp-tetrad had been replaced by phenylalanine to block the final electron transfer step: *D* = −11.2 MHz, *J* = −0.65 MHz. Within the point–dipole approximation, this value of *D* corresponds to a center-to-center separation of 1.91 nm. A subsequent, more refined analysis^14^ of the same data gave slightly different values of the two parameters; however we do not expect these differences to affect the conclusions of the present study. The orientation of TrpH^•+^ relative to FAD^•–^ is that of Trp318 (the third component of the Trp-tetrad) relative to FAD in the crystal structure of pigeon (*Columba livia*) Cry4a,^35^ which has been predicted to closely resemble the structures of other bird Cry4s including Cry4a from the European robin.^36^ The third tryptophan was chosen, rather than the fourth (Trp369), because it seems to make a much larger contribution to the magnetic field effects on wild-type Cry4a.^14,15^ The same values of *D* and *J* were used for the FAD-Z radical pair. The *g*-values of both radicals were taken to be equal to the free-electron value, *g*_e_.

**Figure 1:**
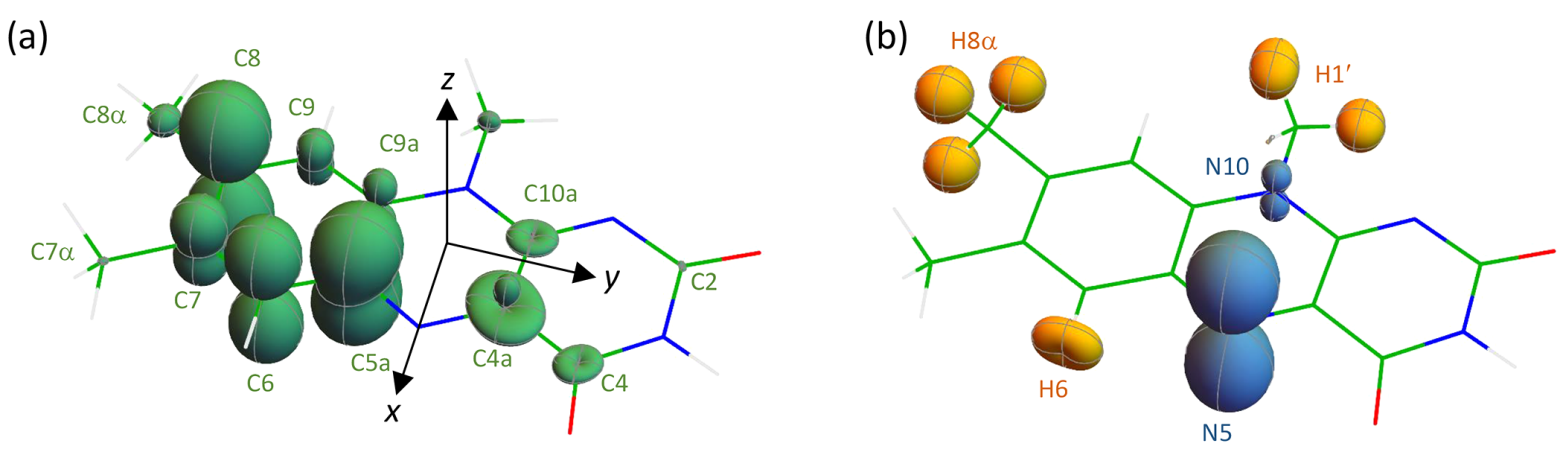
Representations of the hyperfine tensors of (a) ^13^C (green) and (b) ^1^H (yellow) and ^14^N (blue) in FAD^•–^. Nuclei with strongly anisotropic hyperfine interactions have large, nonspherical surfaces, e.g., N5 and C8. Atom labels follow IUPAC nomenclature. The molecular axis system is shown in (a).

^1^H → ^2^H, ^12^C → ^13^C, and ^14^N → ^15^N isotopic substitutions were considered. The natural abundance of ^1^H, ^12^C, and ^14^N in the unsubstituted radicals was taken to be 100% for all three isotopes. The hyperfine tensors for ^1^H, ^13^C, and ^14^N nuclei in FAD^•–^ were calculated using density functional theory (Supporting Information Tables S1–S3).^26^ The corresponding tensors for ^2^H and ^15^N were obtained using the proportionality between the strength of the coupling and the nuclear magnetogyric ratio (γ): γ(^15^N)/γ(^14^N) = −1.402 and γ(^2^H)/γ(^1^H) = 0.154. The flavin component of FAD^•–^ has 11 hydrogens and 4 nitrogens, and TrpH^•+^ has 10 hydrogens and 2 nitrogens. Exact simulations of the spin dynamics of multiple isotopologues of such a large spin system would be prohibitive. Therefore, unless otherwise stated, we chose the seven nuclei in FAD^•–^ with the largest hyperfine interactions: N5, N10, H6, 3 × H8α, and one of the H1′ protons.^26^ The spin system of the TrpH^•+^ radical comprised the electron spin coupled to the indole nitrogen via its strongly anisotropic hyperfine interaction.^26^ See Figure 1 for atom labeling and representations of the FAD^•–^^1^H, ^13^C, and ^14^N hyperfine tensors.

To assess the directional information available from each radical pair, we calculated Φ_S_(θ, ϕ), the fractional yield of the singlet recombination reaction. θ (colatitude) and ϕ (azimuth) define the direction of the magnetic field vector, **B** = |**B**|(sin θ cos ϕ, sin θ sin ϕ, cos θ), relative to the flavin axis system (shown in Figure 1a). **B** is parallel to the *z*-axis when θ = 0 and the *x*-axis when θ = 90°, ϕ = 0. The hyperfine tensor representations in Figure 1 were calculated as follows. The distance from the nucleus in question to the plotted three-dimensional surface in the direction (θ, ϕ) is proportional to **b**^T^·**A**·**b** where **A** is the hyperfine tensor and **b**^T^ = (sin θ cos ϕ, sin θ sin ϕ, cos θ).

In some calculations, a two-site hopping model was used to assess the impact of electron spin relaxation induced by small-amplitude librational motions of the FAD^•–^ within its binding site in the protein. The FAD^•–^ radical was allowed to jump back and forth (with rate constant *k*_m_) between two equally probable orientations, rotated by ±5° around the flavin *x*-axis. The reaction yield was calculated by solving two coupled stochastic Liouville equations, one for each site. Reference (37) gives full details of this calculation.

## Results

### Hydrogen and Nitrogen Isotopologues: Coherent Spin Dynamics

**Figure 2:**
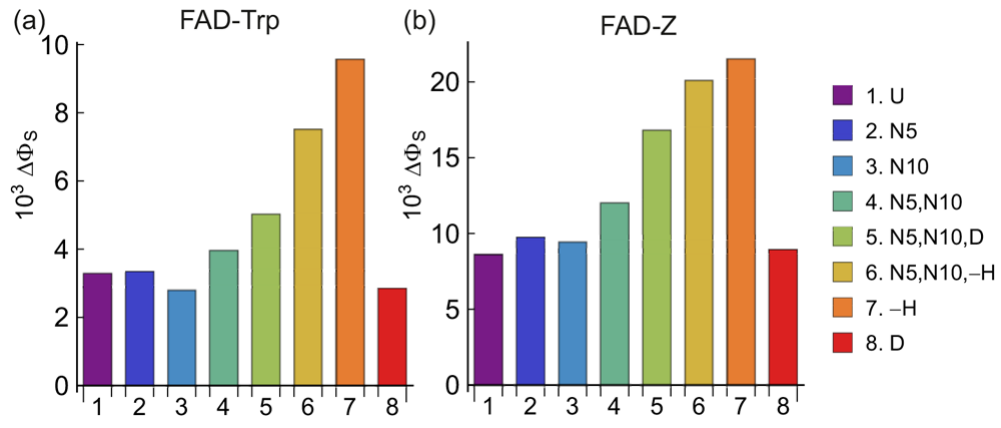
Values of ΔΦ_S_ for nitrogen and hydrogen isotopologues of FAD^•–^ in (a) FAD-Trp and (b) FAD-Z radical pairs. U denotes the unsubstituted radical pair. N5 and N10 denote ^15^N substitution. −H and D indicate that all five hydrogens in FAD^•–^ were omitted or replaced by deuterium, respectively. See Supporting Information Tables S4 and S5 for a summary of the nuclei included in these calculations and a key to the notation.

**Figure 3:**
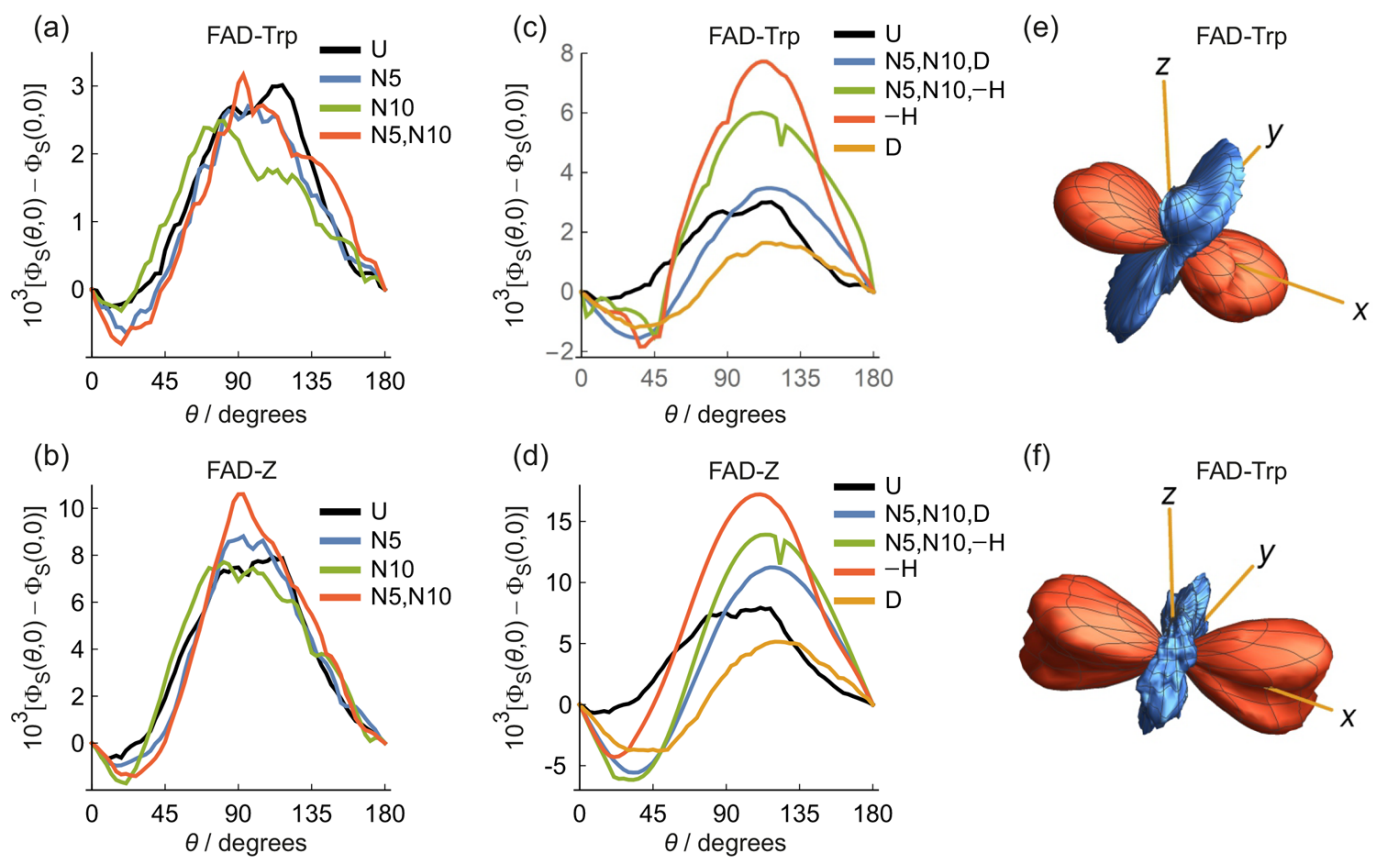
(a–d) Plots of Φ_S_(θ,0) – Φ_S_(0, 0) as a function of magnetic field direction, θ, for nitrogen and hydrogen isotopologues of (a, c) FAD-Trp and (b, d) FAD-Z radical pairs. (e, f) Anisotropy of Φ_S_(θ, ϕ), i.e., Φ̅_S_(θ, ϕ) = Φ_S_(θ, ϕ) – ⟨Φ_S_⟩ where ⟨Φ_S_⟩ is the isotropic component of Φ_S_(θ, ϕ). In (e) and (f), red and blue indicate reaction yields that are, respectively, larger and smaller than the isotropic (i.e., average) reaction yield. (e) FAD-Trp radical pair with two ^14^N and no ^1^H in FAD^•–^ and one ^14^N in TrpH^•+^. Axis lengths = 0.008. (f) FAD-Trp radical pair with two ^14^N and five ^1^H in FAD^•–^ and one ^14^N in TrpH^•+^. Axis lengths = 0.003. The plot labels are the same as in Figure 2. The small dips at θ ≈ 120° in the N5, N10, −H traces in (c) and (d) arise from avoided level crossings. See Supporting Information Tables S4 and S5 for a summary of the nuclei included in these calculations and a key to the notation.

We start by investigating the effects of hydrogen and nitrogen isotopic substitution in the FAD^•–^ component of the FAD-Trp and FAD-Z radical pairs. The results are summarized in Figures 2 and 3. Figure 2a,b shows the anisotropy of the singlet reaction yield, defined as
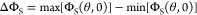
1for several isotopologues. ΔΦ_S_ is regarded as the “signal” that provides the information a bird would need to orient itself in the geomagnetic field: we assume that the bigger the signal, the better the compass sensor. For the same radical pairs, Figure 3a–d plots the dependence of Φ_S_(θ,0) on θ, the direction of the magnetic field vector in the *xz*-plane of FAD^•–^.

The signals for the FAD-Z isotopologues (Figures 2b and 3b,d) are about twice the size of those of FAD-Trp (Figures 2a and 3a,c). Otherwise, FAD-Trp and FAD-Z show broadly similar magnetic isotope effects; we concentrate here on FAD-Trp (Figures 2a and 3a,c). All the ΔΦ_S_ values shown in Figures 2 and 3 are small (∼10^–3^) because of the inclusion of a realistic dipolar interaction (−400 μT) which inhibits the singlet–triplet interconversion caused by the somewhat smaller Zeeman interaction (49 μT).

The first bar in Figure 2a, labeled U, is the FAD-Trp pair without isotopic substitution, i.e., two ^14^N nuclei and five ^1^H in FAD^•–^ and a single ^14^N in TrpH^•+^. The next three bars in Figure 2a show the effects of ^14^N → ^15^N substitution of either N5 alone, N10 alone or N5 and N10 together. The change in ΔΦ_S_ is small. One might have expected a modest increase in ΔΦ_S_ on the basis that the ^14^N hyperfine interactions of the nitrogens at positions 5 and 10 in FAD^•–^ are strongly anisotropic (Figure 1) and increase by 40% on ^15^N substitution. Presumably, any such increase is offset by the smaller spin quantum number of ^15^N (*I* = 1/2) compared to ^14^N (*I* = 1): the magnetic moments  of ^15^N and ^14^N are in the ratio 0.86 to 1 which would be consistent with the small magnetic isotope effects in Figure 2.

The fifth bar in Figure 2a, labeled N5, N10, D, is for a radical pair in which N5 and N10 are ^15^N, and all five hydrogens have been replaced by deuterium. The result is a modest increase in ΔΦ_S_, compared to the unsubstituted case (U). This can be understood in terms of the ∼6.5-fold reduction in the hyperfine interactions on deuteration. Most of the ^1^H interactions in FAD^•–^ are either nearly isotropic (e.g., H8α), small (e.g., H7α), or both (see Figure 1). Deuteration reduces these couplings so that, overall, the flavin radical is magnetically more anisotropic; i.e., the effect of the two anisotropic nitrogens is less diluted by the approximately isotropic hydrogens. This is confirmed by the sixth and seventh bars in Figure 2a: complete removal of the five hydrogens, whether the nitrogens are substituted (N5, N10, −H) or not (−H), results in a further increase in ΔΦ_S_. Deuteration of all five hydrogens in FAD^•–^, without substituting the nitrogens (eighth bar in Figure 2) gave ΔΦ_S_ values comparable to the unsubstituted case.

As well as increasing ΔΦ_S_, deuteration also changes the form of Φ_S_(θ, 0) in a way that ^15^N substitution does not. The four traces in Figure 3a (which show the effects of ^14^N → ^15^N replacement and correspond to the first four bars of Figure 2a) have essentially the same shape, with maxima near θ = 90° and minima around θ = 0°, 180°. The four colored traces in Figure 3c (corresponding to bars 5–8 in Figure 2a), however, have maxima and minima around θ = 110°–125° and θ = 30°–45°, respectively, suggesting that deuterated radical pairs could signal a different compass bearing. This effect is confirmed by Figure 3e,f which shows the anisotropic part of Φ_S_(θ, ϕ) for (e) −H (where both nitrogens are ^14^N and all five hydrogens were omitted) and (f) U (where both nitrogens are ^14^N and all 5 ^1^H are present). Removal of the ^1^H hyperfine interactions rotates the anisotropy by about 30° around the *y*-axis. We anticipate that deuteration would have a similar effect.

### Carbon Isotopologues: Coherent Spin Dynamics

**Figure 4:**
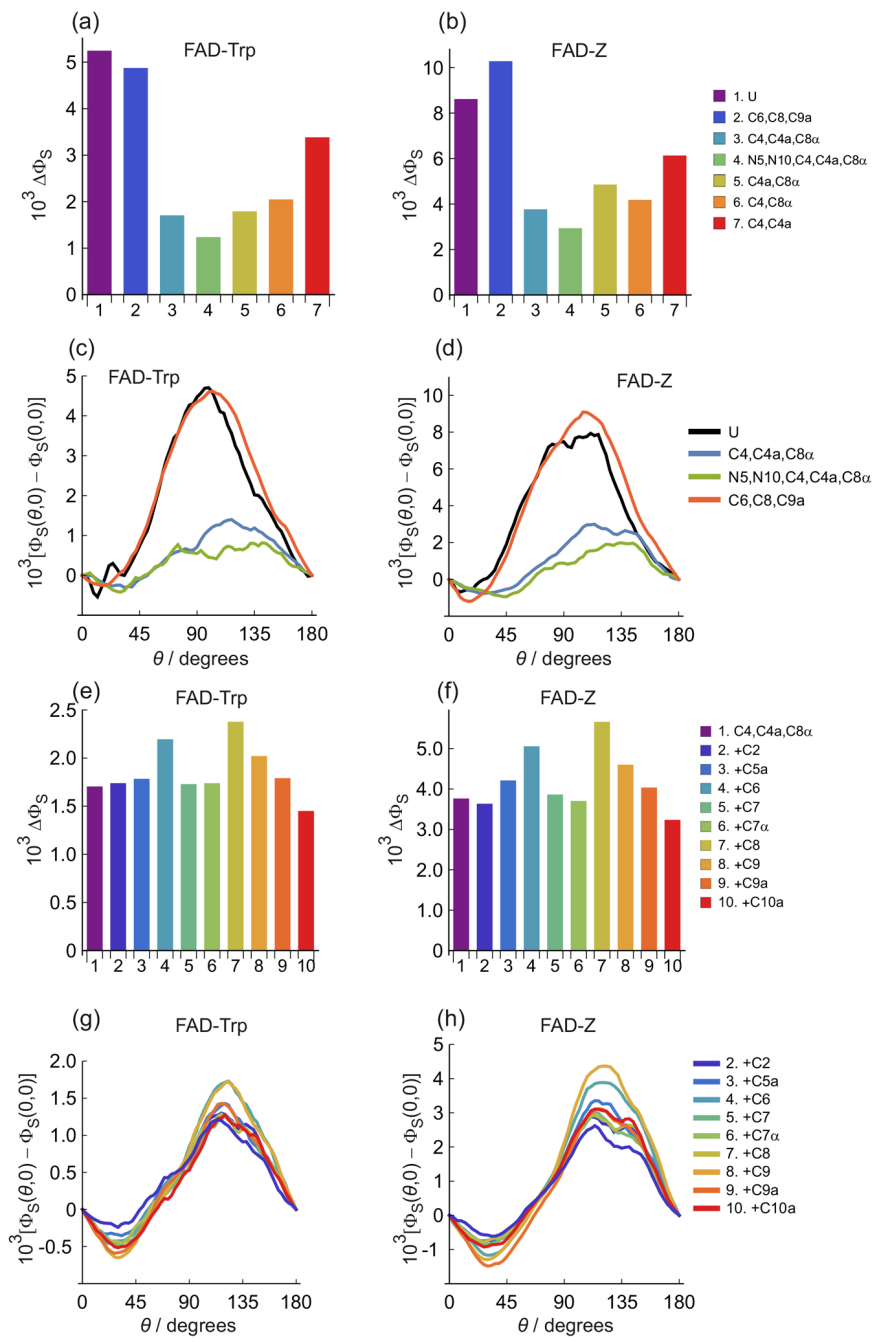
(a, b) Values of ΔΦ_S_ for carbon isotopologues of FAD-Trp and FAD-Z radical pairs, respectively. (c, d) Plots of Φ_S_(θ, 0) – Φ_S_(0, 0) as a function of magnetic field direction, θ, for carbon isotopologues of FAD-Trp and FAD-Z radical pairs, respectively. (e, f) Values of ΔΦ_S_ for carbon isotopologues of FAD-Trp and FAD-Z radical pairs, respectively. (g, h) Plots of Φ_S_(θ, 0) – Φ_S_(0, 0) as a function of magnetic field direction, θ, for carbon isotopologues of FAD-Trp and FAD-Z radical pairs, respectively. The plot and bar chart labels are explained in the text. See Supporting Information Table S4 for a summary of the nuclei included in these calculations.

We now turn to carbon isotopologues with the expectation that replacement of a nonmagnetic nucleus (^12^C) with one that has a magnetic moment (^13^C) might lead to larger changes in ΔΦ_S_ than found for ^1^H → ^2^H or ^14^N → ^15^N substitutions. The 10 ring carbons (C2, C4, C4a, C5a, C6, C7, C8, C9, C9a, C10a) and the two methyl carbons (C7α, C8α) in the isoalloxazine portion of FAD^•–^(Figure 1) were considered. All 220 combinations of three ^12^C → ^13^C substitutions were simulated for both FAD-Trp and FAD-Z. Some of the results are summarized in bar-chart form in Figure 4a,b and as a function of θ in Figure 4c,d. In both cases, the hyperfine interaction of the H1′ proton in FAD^•–^ in FAD-Trp was omitted to reduce the size of the calculation.

Once again, the results for FAD-Z (Figure 4b,d,f,h) were larger by a factor of ∼2 but otherwise similar to those for FAD-Trp (Figure 4a,c,e,g). Focusing now on the bar chart for FAD-Trp (Figure 4a), none of the 220 carbon isotopologues gave a value of ΔΦ_S_ larger than the unsubstituted case (U, the first bar in Figure 4a). The combination that came closest to the unsubstituted radical pair was C6, C8, C9a (second bar). Even though these carbons have strongly anisotropic hyperfine tensors, with the same symmetry as N5 and N10 (large *z*-component, small *x*- and *y*-components, Figure 1), they do not enhance the anisotropy of the magnetic field effect, which appears to be largely “saturated” by the effects of N5 and to a lesser extent N10.

The combination of three carbons that produced the smallest ΔΦ_S_ (3.1 times smaller than the unsubstituted case, U) was C4, C4a, C8α (third bar in Figure 4a). ΔΦ_S_ is smaller still (4.2 times smaller than U) if, in addition, N5 and N10 are replaced by ^15^N (fourth bar). C4, C4a, and C8α are among the carbons with the largest hyperfine components in the *xy*-plane of the flavin (Figure 1). The large *xy*-components of these nuclei could reduce ΔΦ_S_ by offsetting the effect of the large *z*-components of N5 and N10, i.e., by making FAD^•–^ less anisotropic overall.

A smaller reduction in ΔΦ_S_ (compared to the unsubstituted case) is seen when any two of C4, C4a, and C8α are substituted (bars 5–7 in Figure 4a). Replacing a fourth ^12^C by ^13^C, in addition to C4, C4a, and C8α, gave neither a further reduction in ΔΦ_S_ (Figure 4e) nor much of a change in the θ-dependence (Figure 4g).

Smaller reductions in ΔΦ_S_ were found for other combinations of three carbons. Table 1 shows the ten “best” sets (best at reducing ΔΦ_S_), starting with C4, C4a, C8α and working down. All but one of the ten sets contain two of C4, C4a, and C8α. C8α was present in all ten, C4a in six, and C4 in four. The reductions in ΔΦ_S_, relative to the unsubstituted case, varied between 2.9-fold (set 2) and 2.6-fold (set 10) compared to 3.1 for C4, C4a, C8α (set 1).

**Table 1:** Ten Sets of Three ^12^C → ^13^C Substitutions That Give the Biggest Reductions in ΔΦ_S_^a^

	C2	C4	C4a	C7	C7α	C8α	C9a	C10a	10^3^ ΔΦ_S_
1		•	•			•			1.70
2	•		•			•			1.81
3		•				•		•	1.85
4			•			•	•		1.91
5			•			•		•	1.94
6			•	•		•			1.96
7			•		•	•			1.97
8		•				•	•		1.98
9						•	•	•	1.99
10		•		•		•			2.00

a The largest reduction was found for C4, C4a, C8α. The final column gives values of the reaction yield anisotropy.

### Nitrogen Isotopologues: Spin Relaxation

None of the calculations reported above included spin relaxation, a process that is unlikely to be negligible *in vivo* and is expected to attenuate the magnetic field effects that arise from the coherent spin dynamics.^38−41^ To explore the magnetic isotope effect on the loss of spin coherence, we modeled the librational motion of the FAD radical in its binding site in a protein by allowing it to wobble back and forth between two equally probable orientations, rotated by ±5° around the flavin *x*-axis.^37^ Very similar results were found when this rocking motion was around the *y*-axis. Little relaxation is expected either in the motional narrowing limit, when the rate constant *k*_m_ for the wobbling motion is much larger than the hyperfine interactions, or in the static limit in which *k*_m_ is much smaller than the hyperfine interactions. It is when *k*_m_ is comparable to the hyperfine interactions, i.e., 10^7^ s^–1^ < *k*_m_ < 10^8^ s^–1^, that the spin relaxation should be most effective at destroying the coherence on which the magnetic sensitivity relies.

**Figure 5:**
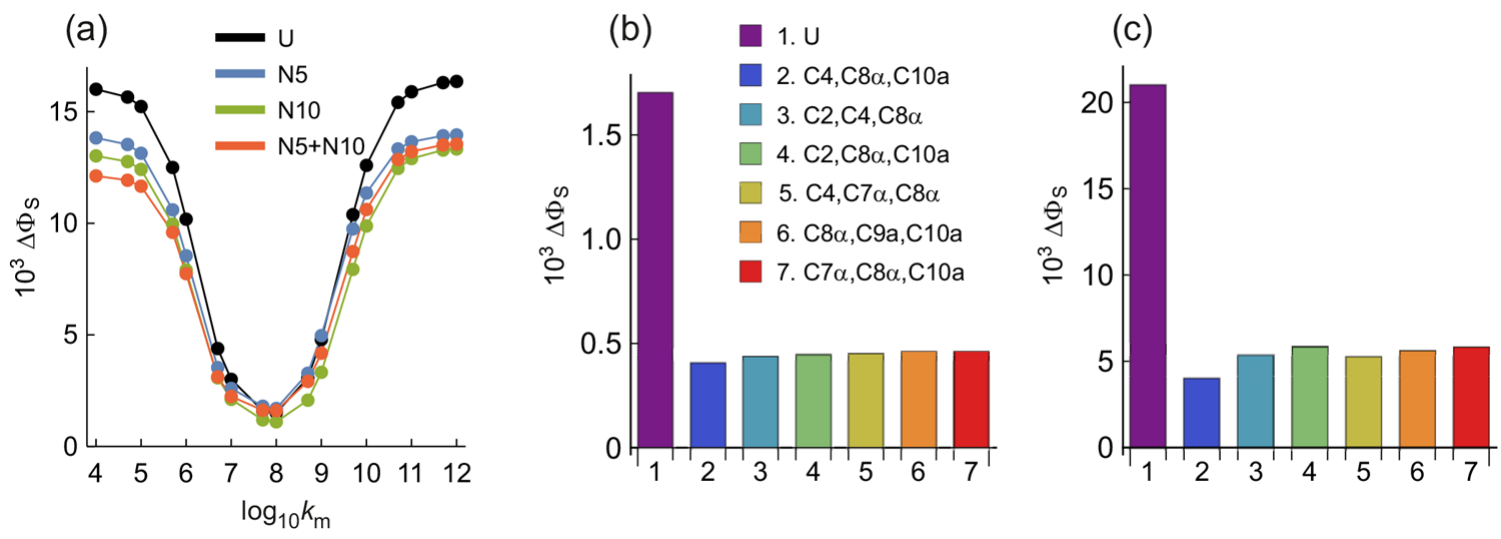
(a) Plots of ΔΦ_S_ for nitrogen isotopologues as a function of the rate constant for the wobbling motion of FAD^•–^ in a FAD-Z radical pair. (b, c) Values of ΔΦ_S_ for carbon isotopologues of FAD-Z radical pairs with and without spin relaxation, respectively. The plot and bar chart labels are explained in the text. See Supporting Information Table S4 for a summary of the nuclei included in these calculations.

Figure 5a shows the dependence of ΔΦ_S_ on *k*_m_ for unsubstituted (black) and ^14^N → ^15^N substituted (color) FAD-Z radical pairs. Similar results are expected for FAD-Trp. ΔΦ_S_ for the unsubstituted radical pair (U) is reduced 11-fold from 0.0160 when *k*_m_ = 10^4^ s^–1^ to 0.0015 when *k*_m_ = 10^8^ s^–1^. Replacing either N5 or N10 or both by ^15^N reduced ΔΦ_S_ for all but the *k*_m_ values (10^7^–10^8^ s^–1^) that induce the fastest relaxation. Given that the difference between the unsubstituted radical pair and the ^15^N isotopologues in Figure 5a is only present for very slow and very fast motions, where spin relaxation is ineffective, it seems that the majority of the ^14^N → ^15^N effect arises from the coherent spin dynamics (shown in Figure 2) rather than this form of spin relaxation.

### Carbon Isotopologues: Spin Relaxation

We anticipate that replacement of nonmagnetic ^12^C nuclei by ^13^C nuclei that have strongly anisotropic hyperfine interactions (Figure 1) could enhance spin relaxation effects and lead to bigger differences between substituted and unsubstituted radical pairs. To keep the computational demands within reasonable bounds, FAD-Z was modeled for a single value of *k*_m_ (= 10^8^ s^–1^, chosen to give a large change in ΔΦ_S_) with just four hyperfine interactions in FAD^•–^ (N5, N10, H6 and one of the H8α protons). As above, all 220 three-carbon substitutions were considered.

Data for the five sets of three carbons that produce the largest reductions in ΔΦ_S_ compared to the unsubstituted case (bar 1) are shown as bars 2–7 in Figure 5b. All six attenuate the signal by ∼75%. Similar reductions were found when spin relaxation was not included (Figure 5c). The similarity of Figure 5b,c suggests that the spin relaxation induced by the librational motion of FAD^•–^ is dominated by the modulation of the ^14^N hyperfine tensors.

### Magnitude of ΔΦ_S_

Finally in this section, we comment briefly on the strength of the signal (ΔΦ_S_) assumed to allow a bird to orient in the geomagnetic field. In all of the simulations presented here, ΔΦ_S_ is of the order of 10^–3^, corresponding to a ∼0.1% change in the reaction yield for a ∼90° change in orientation with respect to the ∼50 μT magnetic field. One might reasonably ask whether such a small magnetic field effect is sufficient to form the basis of a viable compass magnetoreceptor. An answer to this important question will have to wait until more is known about the structure, binding partners, and signaling of cryptochromes *in vivo*. We have so little knowledge of factors such as spin relaxation, amplification mechanisms, spatial and temporal integration of information from receptors distributed around the retina, and so on that it is impossible to say how big ΔΦ_S_ would need to be. Magnetic field effects at the level of 0.1% are undeniably small but if cryptochromes really are the magnetoreceptors, Nature must have found a way to cope with weak signals.

## Discussion and Conclusions

The dominant effect of isotopic substitution on the spin dynamics of radical pairs is to scale the strength of the hyperfine interactions and thereby alter the sensitivity to external magnetic fields. In the context of magnetoreception, the relevant quantities are the variation of the reaction yield, Φ_S_(θ, ϕ), with the direction of a ∼50 μT magnetic field and the magnitude of this anisotropy, ΔΦ_S_ (eq 1). In the calculations reported here, we explored the effects of replacing ^1^H by ^2^H, ^12^C by ^13^C, and ^14^N by ^15^N, for both of the candidate radical pairs in cryptochrome: FAD-Trp and FAD-Z.

Broadly similar magnetic isotope effects were found for FAD-Trp and FAD-Z, with FAD-Z having ΔΦ_S_ values about twice those of FAD-Trp, other things being equal (Figures 2–4). The difference between the two radical pairs is less pronounced than reported by Lee et al.^26^ because of the inclusion of a realistic dipolar coupling.^42^

Nitrogen substitution does not produce large changes in either Φ_S_(θ, ϕ) or ΔΦ_S_ whether spin relaxation arising from librational modulation of the hyperfine interactions is included or not (Figures 2 and 5a). This is consistent with the similarity of the magnetic moments of the two isotopes (^15^N:^14^N = 0.86:1.00).

Replacement of hydrogen by deuterium, which scales hyperfine couplings by a factor of 0.154 and the nuclear magnetic moment by 0.251, has the effect of increasing ΔΦ_S_ and changing the shape of Φ_S_(θ, ϕ) (Figure 3). This seems to occur because the approximately isotropic ^1^H hypefine interactions dilute the contributions of N5 and N10 to ΔΦ_S_, an effect that is diminished by deuteration.^26^ Spin relaxation of the deuterated FAD^•–^ radical was not investigated. Recalling the quadratic dependence on the hyperfine coupling strength,^43^ spin relaxation arising from modulation of ^2^H hyperfine couplings should be [γ(^2^H)/γ(^1^H)]^2^ = 42 times slower than that from the corresponding ^1^H hyperfine couplings.

The set of three ^12^C → ^13^C substitutions that produces the largest reduction in ΔΦ_S_ is C4, C4a, and C8α (Figure 4). This reduction is smaller when only two of C4, C4a, and C8α are replaced or when another carbon is added to this set of three. These three carbons feature strongly in the 10 best sets (Table 1).

**Figure 6:**
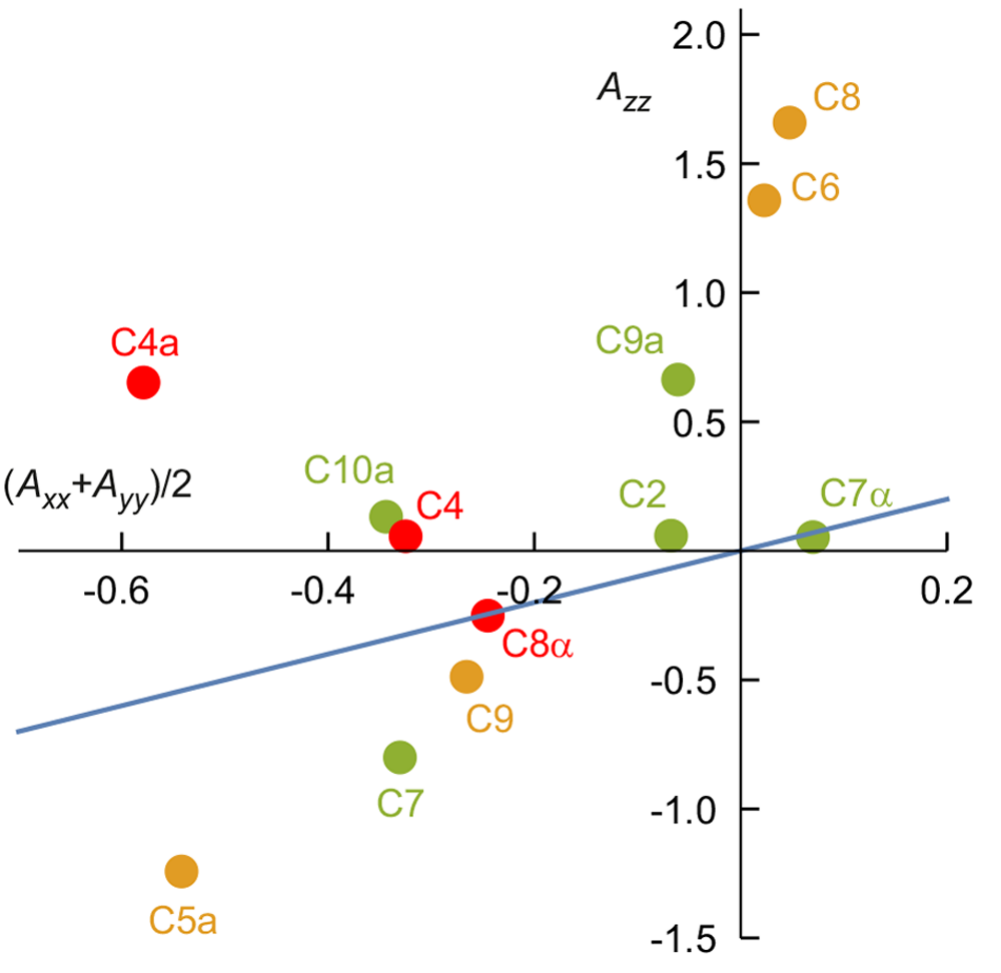
Principal hyperfine tensor components for the 12 carbons in the flavin portion of FAD^•–^. The color code is explained in the text. The blue line is *A*_*zz*_ = (*A*_*xx*_ + *A*_*yy*_)/2.

It is not clear why C4, C4a, C8α have the largest effect on ΔΦ_S_; a clue comes from the scatter plot in Figure 6 in which the principal components (*A*_*xx*_, *A*_*yy*_, *A*_*zz*_) of the ^13^C hyperfine interactions are shown in red for C4, C4a, and C8α, in green for the carbons that feature in the top 10 sets in Table 1, and in orange for the rest. Broadly speaking, it is the carbons with small |*A*_*zz*_| and large |*A*_*xx*_ + *A*_*yy*_|/2 that have the greatest effect on ΔΦ_S_. Inclusion of these ^13^C atoms seems to offset the anisotropic effects of N5 and N10, which both have large |*A*_*zz*_| ≫ |*A*_*xx*_ + *A*_*yy*_|/2, and so reduce the overall magnetic anisotropy.

Two main conclusions emerge from this work. (1) Perdeuteration of FAD appears to be the best way to boost the reaction yield anisotropy and to change its symmetry. (2) ^13^C substitution of C4, C4a, and C8α, or one of the other nine combinations in Table 1 seems to be the best way of reducing the reaction yield anisotropy. Both predictions will be tested by measuring magnetic field effects on cryptochromes with the appropriate FAD isotopologues incorporated.

## Supporting Information Available

Hyperfine coupling tensors, lists of nuclei included in calculations shown in Figures 2–5, and notation used in Figures 2 and 3 (PDF)
