# Supplementary material for: Isotope Substitution Effects on the Magnetic Compass Properties of Cryptochrome-Based Radical Pairs: A Computational Study
Source: J Phys Chem B. 2023 Jan 20;127(4):838–45. doi: 10.1021/acs.jpcb.2c05335 (PMC9900586; doi:10.1021/acs.jpcb.2c05335)
Supplement: Supplementary file 1 — jp2c05335_si_001.pdf [file jp2c05335_si_001.pdf]

Isotope Substitution Effects on the Magnetic Compass Properties of Cryptochrome-Based Radical Pairs: a Computational Study

[1]Gediminas Jurgis Pažėra, [1]Philip Benjamin, [2,3]Henrik Mouritsen and [1]P. J. Hore*

[1]Department of Chemistry, University of Oxford, Oxford OX1 3QZ, UK

[2]Institut für Biologie und Umweltwissenschaften, Carl-von-Ossietzky Universität Oldenburg, Oldenburg 26111, Germany

[3]Research Centre for Neurosensory Science, University of Oldenburg, Oldenburg 26111, Germany

* Author for correspondence: peter.hore@chem.ox.ac.uk

nucleus	hyperfine tensor / mT
^{14}N5	$\begin{pmatrix} -0.0995 & 0.0029 & 0 \\ 0.0029 & -0.0875 & 0 \\ 0 & 0 & 1.7569 \end{pmatrix}$
^{14}N10	$\begin{pmatrix} -0.0149 & 0.0021 & 0 \\ 0.0021 & -0.0237 & 0 \\ 0 & 0 & 0.6046 \end{pmatrix}$
^{1}H6	$\begin{pmatrix} -0.2009 & -0.0328 & 0 \\ -0.0328 & -0.5271 & 0 \\ 0 & 0 & -0.4336 \end{pmatrix}$
^{1}H8	$\begin{pmatrix} 0.4313 & 0.0369 & 0 \\ 0.0369 & 0.4786 & 0 \\ 0 & 0 & 0.4100 \end{pmatrix}$
^{1}H1′	$\begin{pmatrix} 0.3492 & -0.0015 & 0 \\ -0.0015 & 0.2459 & 0 \\ 0 & 0 & 0.2289 \end{pmatrix}$

Table S1. ^{1}H and ^{14}N hyperfine tensors for $FAD^{\bullet -}$. Calculated by Dr Ilya Kuprov (University of Southampton) using density functional theory in Gaussian-03 at the UB3LYP/EPR-III level. The calculation was done for the radical anion of 7,8,10-trimethyl isoalloxazine (lumiflavin) *in vacuo*. The atom numbering scheme is as shown in Fig. 1.

nucleus	hyperfine tensor / mT
^{13}C2	$\begin{pmatrix} -0.0816 & -0.0186 & 0 \\ -0.0186 & -0.0534 & 0 \\ 0 & 0 & 0.0589 \end{pmatrix}$
^{13}C4	$\begin{pmatrix} -0.3217 & 0.0302 & 0 \\ 0.0302 & -0.3279 & 0 \\ 0 & 0 & 0.0564 \end{pmatrix}$
^{13}C4a	$\begin{pmatrix} -0.5995 & -0.0463 & 0 \\ -0.0463 & -0.5583 & 0 \\ 0 & 0 & 0.6520 \end{pmatrix}$
^{13}C5a	$\begin{pmatrix} -0.5851 & 0.0614 & 0 \\ 0.0614 & -0.4989 & 0 \\ 0 & 0 & -1.2420 \end{pmatrix}$
^{13}C6	$\begin{pmatrix} 0.0204 & -0.0004 & 0 \\ -0.0004 & 0.0251 & 0 \\ 0 & 0 & 1.3577 \end{pmatrix}$
^{13}C7	$\begin{pmatrix} -0.3265 & 0.0165 & 0 \\ 0.0165 & -0.3341 & 0 \\ 0 & 0 & -0.8004 \end{pmatrix}$
^{13}C7α	$\begin{pmatrix} 0.0667 & -0.0061 & 0 \\ -0.0061 & 0.0733 & 0 \\ 0 & 0 & 0.0531 \end{pmatrix}$
^{13}C8	$\begin{pmatrix} 0.0393 & 0.0122 & 0 \\ 0.0122 & 0.0555 & 0 \\ 0 & 0 & 1.6589 \end{pmatrix}$
^{13}C8α	$\begin{pmatrix} -0.2520 & 0.0134 & 0 \\ 0.0134 & -0.2383 & 0 \\ 0 & 0 & -0.2511 \end{pmatrix}$
^{13}C9	$\begin{pmatrix} -0.2846 & 0.0015 & 0 \\ 0.0015 & -0.2468 & 0 \\ 0 & 0 & -0.4877 \end{pmatrix}$
^{13}C9a	$\begin{pmatrix} -0.0711 & 0.0074 & 0 \\ 0.0074 & -0.0503 & 0 \\ 0 & 0 & 0.6632 \end{pmatrix}$
^{13}C10a	$\begin{pmatrix} -0.3215 & 0.0079 & 0 \\ 0.0079 & -0.3660 & 0 \\ 0 & 0 & 0.1320 \end{pmatrix}$

Table S2. ^{13}C hyperfine tensors for $FAD^{\bullet -}$. For other details, see Table S1.

nucleus	hyperfine tensor / mT
^{14}N1	$\begin{pmatrix} 0.3176 & 0.4962 & -0.2092 \\ 0.4962 & 0.5919 & -0.2662 \\ -0.2092 & -0.2662 & 0.0551 \end{pmatrix}$

Table S3. ^{14}N hyperfine tensor for $\mathrm{TrpH}^{\bullet +}$. The calculation was done for the radical cation of tryptophan *in vacuo*. N1 is the indole nitrogen. For other details, see Table S1.

	FAD radical
Fig. 2, Fig. 3a-d	N5, N10, H6, 3×H8α, 1× H1′<br>except for –H and N5,N10, –H which were N5, N10
Fig. 3e	N5, N10
Fig. 3f	N5, N10, H6, 3×H8α
Fig. 4	N5, N10, H6, 3×H8α + 2, 3, or 4 carbons
Fig. 5a	N5, N10, H6, 3×H8α
Figs 5b,c	N5, N10, H6, 1×H8α + 3 carbons

Table S4. Nuclei included in the model $\mathrm{FAD}^{\bullet -}$ radical for the calculations shown in Figs 2-5.

	label	N5 isotope	N10 isotope	hydrogen isotopes
1	U	14	14	H
2	N5	15	14	H
3	N10	14	15	H
4	N5,N10	15	15	H
5	N5,N10,D	15	15	D
6	N5,N10,–H	15	15	none
7	–H	14	14	none
8	D	14	14	D

Table S5. Notation used in Figs 2 and 3.
